# The Immigrant Mortality Advantage Among Over-65 Middle Eastern and North African Immigrants to the United States

**DOI:** 10.1007/s10903-024-01622-5

**Published:** 2024-08-04

**Authors:** Elyas Bakhtiari, Jacinta Das

**Affiliations:** 1 Department of Sociology, William and Mary, Williamsburg, VA 23185 USA; 2 William and Mary, Williamsburg, VA 23185 USA

**Keywords:** Immigrant health, Middle Eastern and North African, Immigrant mortality advantage, Over-65 mortality

## Abstract

Immigrants to the United States often have longer life expectancies than their U.S.-born counterparts, however it is unclear whether a similar “immigrant advantage” exists for immigrants from the Middle East and North Africa (MENA). This study uses a novel machine-learning name classifier to offer one of the first national-level examinations of MENA mortality patterns by nativity in the United States. A recurrent neural network model was developed to identify MENA individuals based on given name and surname characteristics. The model was trained on more than 2.5 million mortality-linked social security records in the Berkeley Unified Numident Mortality Database (BUNMD). Mortality rates and life expectancy were estimated using a Gompertz distribution and maximum likelihood estimation, focusing on high-coverage years between 1988 and 2005 and deaths over age 65. Foreign-born MENA men over 65 showed a significant immigrant mortality advantage with a hazard ratio (HR) of 0.64 and an estimated 3.13 additional years of life expectancy at age 65 compared to U.S.-born counterparts. Foreign-born MENA women also exhibited an advantage, with a HR of 0.71 and an additional 2.24 years of life expectancy at age 65. This study is one of the first national-level analyses of mortality outcomes among the over-65 MENA population in the United States, finding a MENA immigrant mortality advantage. The results suggest further research is needed to identify and disaggregate the MENA population in health research.

**Supplementary Information:**

The online version contains supplementary material available at 10.1007/s10903-024-01622-5.

## Introduction

Immigrant populations tend to have better health and longer life expectancies than their non-immigrant counterparts [1–4]. This phenomenon—sometimes referred to as a “paradox” because it persists despite other risk factors among immigrant populations—tends to diminish with duration of residence and across generations, resulting in worse health and mortality profiles for second-plus generation co-ethnics relative to their foreign-born counterparts [5, 6]. Although this “immigrant advantage” in health and mortality has been observed among most major immigrant populations to the United States, some research suggests immigrants from the Middle East and North Africa (MENA) may be an exception.

A few studies focused on Arab Americans have found the opposite of the expected immigrant advantage pattern, in which foreign-born Arabs report worse self-rated health [7], functional limitations [8, 9], cognitive disability [10, 11], or psychosocial wellbeing [12, 13] than their U.S.-born counterparts. Other research has observed no significant differences between foreign-born and U.S.-born Arab or MENA populations [14–16]. A tangential body of literature has examined Arab or MENA population health without explicitly comparing nativity status, finding Arab and MENA populations may have comparable or worse outcomes than non-Hispanic whites [17, 18]. The few studies that have examined mortality as an outcome among MENA populations have used either California or Michigan vital records, and found mixed results and possible variation by gender and age [19–21]. A study that explicitly examined nativity differences in cause-specific mortality in California found variation that did not neatly align with the immigrant advantage: MENA immigrants had higher odds of mortality from colorectal cancer, diabetes, and heart disease, but lower odds from COPD and suicide, with variation in outcomes by gender [22].

The mixed results among studies reflect the sparse data available for studying Arab and MENA populations. Many sources of data collection rely on ethnic and racial identity categories similar to the U.S. Census, which includes individuals with ancestry from the Middle East and North Africa in its definition of the white racial category. As a result, many data sources do not explicitly identify individuals of MENA background for study, although a growing body of literature suggests their experiences of racialization and discrimination warrant more attention as risk factors for health [23–26]. Previous research has often addressed this issue by relying on state-specific datasets with identifying information, particularly from Michigan and California which have large MENA populations [14, 16, 17, 19, 26–28], or on nationally-representative surveys that rely on self-reported measures of ancestry and have relatively small MENA subsamples [12, 15, 29, 30].

In the absence of reliable data on ancestry, religion, and ethnicity in many official records, name-matching algorithms have proven effective in identifying such populations [19, 26, 28, 31, 32]. This approach relies on matching target name data with a source list of names to estimate an individual’s ethnicity or origin country. For instance, researchers have used the Social Security Administration’s file of applications for social security cards, approximately 420 million records dating back to 1936, as a master list to match and classify names in a target dataset [32]. This technique requires an exact match and may not detect spelling variants, which can be a particular problem for names transliterated from another language.

Our study innovates by developing a machine-learning algorithm for classifying individuals with likely ancestry from the Middle East and North Africa (MENA) using name characteristics, rather than full name matches. By training a long short term memory recurrent neural network model on a set of foreign-born MENA training names, our method can be trained on an internal subset of data to probabilistically predict MENA background using common linguistic and textural characteristics of names common to MENA countries. We apply this method to more than 4.3 million records in the Berkeley Unified Numident Mortality Database, a collection of social security records that contains both given name and surname information, as well as year of death. We then estimate over-65 mortality using high-coverage social security death records in the database, and test for between-group relative mortality inequalities and differences by nativity status. To our knowledge, this is the first national-level examination of mortality differences by nativity among the U.S. MENA population.

## Methods

### Data

This study examines over-65 mortality by nativity using the Berkeley Unified Numident Mortality Database, a dataset containing forty-nine million social security records with demographic and other information released by the National Archives [33]. Each record represents an individual, with information compiled from social security applications, claims, and death files linked by Social Security numbers. The dataset includes each individual’s given name, surname, race at last application, and place of birth. The earliest death year of an individual in the dataset was 1899, and the most recent death year was 2007. However, we rely on a subsample with near-complete coverage of deaths between the years 1988 and 2005, based on comparisons with the Human Mortality Database [33]. This research was approved by the William and Mary Protection of Human Subjects Committee.

### Measures and Methods

The given name and surname information in the BUNMD dataset allows for identifying MENA populations using probabilistic name categorization techniques. One common approach in the public health literature relies on identifying ethnically-unique names by matching study records to an externally-generated list of names common to a country, region, or ethnicity [19, 26, 28, 31, 32]. Although this method effectively identifies the most ethnically-distinct names, it may miss spelling variants or less-common names among the full population.

Our study addresses these limitations by developing a machine-learning recurrent neural network model that can classify names without a direct match and can be trained internally within a large dataset. We train a long short-term memory (LSTM) recurrent neural network on the textual characteristics of each given name and surname combination in order to identify characteristics of ethnically-distinct names, adopting a method previously developed to classify ethnicity and race using name lists from voter registration data and Wikipedia [34].^1^ By converting each full name into variable-length character clusters common in the dataset, a process known as byte pair encoding, the LSTM model can detect both character clusters that may be unique to a given ethnic group or language, such as unique consonant clusters common to Arabic or Persian names (e.g., “kh”), and sequences of letters that may be common in spelling variations of the same name. These textual characteristics can predict name similarity even for names without a direct spelling match.^2^

The training dataset was created using a foreign-born subset of the BUNMD data, with MENA records identified by place of birth in a MENA country. The names of an individual’s mother and father were added to the dataset to expand the training data. Records of individuals who last reported racial identity prior to 1981 were excluded, because the SSA relied on a three-category racial classification system that only included Black, White, and Other categories. This resulted in 2,590,329 total training names, of which 82,476 were classified as MENA.

For individuals born in MENA countries, the algorithm had a precision of 0.86, recall of 0.79, and an F1-score of 0.82 on a split test dataset. Additional details about the name-classification method can be found in the accompanying methodological appendix.

### Measuring Mortality

The BUNMD dataset contains the age of death for each record. But because full cohort mortality data is not available for most cohorts in the BUNMD data, truncation of both left and right tails can bias estimates in conventional linear regression models. Our mortality analysis instead relies on a method developed by Goldstein et al. [35] for modeling partially observed cohort data using maximum likelihood estimation and a Gompertz distribution of mortality rates. The approach models the age-specific hazard, x, in which:

$${h}_{i}\left(x|\beta \right)={a}_{0}{e}^{{b}_{0}x}{e}^{\beta {Z}_{i}}$$

The hazard is estimated based on a baseline level of mortality, *a*_0_, a rate of increase of mortality, b_0_, and a set of parameters *β* and covariates *Z*_*i*_.

Because the deaths are truncated, we limit the analysis to high coverage years after 1988 and before 2005, and focus on high coverage ages of death over 65. Analyses are weighted using a post-stratification person-weight to Human Mortality Database totals. In a supplemental analysis in the Appendix, we conducted similar analysis using linear regressions on death age that include birth year fixed effects to account for variation in truncation across birth cohorts, as recommended by Goldstein and Breen [33].

## Results

**Fig. 1:**
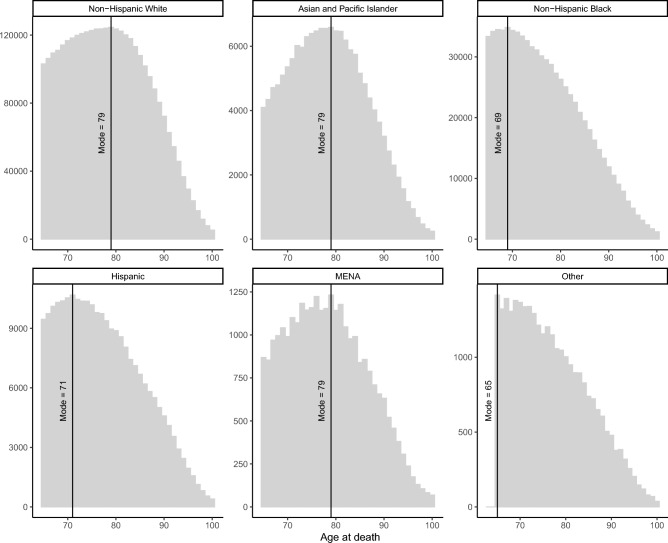
Histograms of age at death by ethno-racial group for deaths between 1988 and 2005. Distributions reflect a high-coverage subsample of deaths that occurred between 1988 and 2005 for individuals 65 and over

Although most individuals classified as MENA were previously identified as either non-Hispanic white (85%) or Asian (13%) in the social security records, our initial comparison suggests their over-65 life expectancy patterns are distinct from either group. Figure 1 depicts the truncated distributions of death ages for each of the major ethno-racial groups, with MENA populations identified using the name-classification algorithm. Although the modal age at death was similar for MENA, Asian, and non-Hispanic White populations, the shape of each distribution differed, as did the mean age of death and other descriptive characteristics.

Table 1 presents the descriptive statistics of the BUNMD sample, broken down by nativity status of each group. The aggregate distribution of mortality for the MENA population is shaped by its large proportion of immigrants. Roughly 61% of the MENA records in the BUNMD files were born in a MENA country, which is comparable to the foreign-born proportion of the over-65 MENA population in the 2000 Census [36]. It is worth noting that this is higher than the proportion of the overall MENA population that is foreign-born, because the over-65 sample captures early waves of immigrants and fewer second-plus generation MENA Americans who are more likely to be younger than 65. The descriptive statistics suggest the possibility of an immigrant mortality advantage for men but a possible disadvantage for women, as both the mean and modal age at death for foreign-born MENA women was lower than that of their U.S.-born MENA counterparts.

**Table 1:** Descriptive statistics

	Nativity	n	Age at death (mean)	Age at death (mode)	Mean year of birth
**Men**					
Non-Hispanic White	US-born	1,393,817	76.7	71	1922
	Foreign-born	87,322	79.5	80	1919
Asian and Pacific Islander	US-born	21,421	78.0	77	1920
	Foreign-born	59,375	77.8	77	1921
Non-Hispanic Black	US-born	368,408	75.5	66	1922
	Foreign-born	10,084	76.0	70	1923
Hispanic	US-born	60,028	75.4	70	1923
	Foreign-born	64,544	76.9	71	1922
MENA	US-born	6164	77.0	69	1921
	Foreign-born	9418	77.8	75	1921
Other	US-born	13,129	75.6	69	1922
	Foreign-born	1617	77.2	77	1920
**Women**					
Non-Hispanic White	US-born	1,599,634	79.9	82	1918
	Foreign-born	117,292	82.5	88	1916
Asian and Pacific Islander	US-born	18,341	80.9	85	1917
	Foreign-born	54,167	79.0	80	1920
Non-Hispanic Black	US-born	357,680	78.1	69	1920
	Foreign-born	12,077	78.6	77	1921
Hispanic	US-born	52,039	77.5	72	1921
	Foreign-born	67,250	79.7	79	1920
MENA	US-born	4872	79.9	79	1918
	Foreign-born	7973	79.3	76	1920
Other	US-born	12,870	77.5	67	1921
	Foreign-born	1651	79.0	76	1919

Data come from the Berkeley Unified Numident Mortality Database of social security records. A subsample of high-coverage deaths between 1988 and 2005 was selected for analysis

However, because the average age at death is calculated from partial and doubly-truncated death distributions, descriptive comparisons may not capture the actual between-group differences along the distribution. The average birth year for foreign-born MENA women was earlier in the sample, which can affect the portion of a cohort death distribution being observed. The Gompertz maximum likelihood estimation models offer a better estimate of the nativity differences for each group. Table 2 presents nativity differences by sex for each ethno-racial group in the sample, both as hazard ratios and an estimated difference in life expectancy between foreign-born and U.S.-born members of each group.

**Table 2:** Estimates of nativity differences in mortality by ethno-racial group

	Hazard ratio			Effect on e65		
	Estimate	Lower	Upper	Estimate	Lower	Upper
**Men**						
Non-Hispanic White	0.85	0.84	0.86	1.16	1.10	1.22
Asian and Pacific Islander	0.75	0.73	0.77	2.03	1.86	2.21
Non-Hispanic Black	0.95	0.92	0.98	0.38	0.13	0.63
Hispanic	0.72	0.70	0.73	2.43	2.28	2.59
MENA	0.64	0.61	0.68	3.13	2.73	3.52
Other	1.01	0.94	1.09	− 0.07	− 0.64	0.51
**Women**						
Non-Hispanic White	0.88	0.87	0.89	0.86	0.79	0.92
Asian and Pacific Islander	0.88	0.85	0.91	0.88	0.64	1.11
Non-Hispanic Black	0.65	0.61	0.69	3.39	2.90	3.87
Hispanic	0.67	0.65	0.69	2.61	2.41	2.80
MENA	0.71	0.66	0.77	2.24	1.74	2.74
Other	1.14	1.03	1.26	− 0.99	− 1.77	− 0.19

Estimates reflect the difference between foreign-born and U.S.-born individuals within each ethno-racial group. The first three columns estimate the hazard ratio of foreign-born nativity status relative to U.S.-born. The last three columns convert this difference into an estimated effect on life expectancy conditional on living to age 65

When looking at truncated Gompertz estimates, evidence for an immigrant mortality advantage is clearer. For MENA men, the hazard ratio for foreign-born men was 0.64 (CI 0.61–0.68), which corresponds to an additional 3.13 years of life expectancy at age 65, relative to U.S.-born MENA men. This was the largest nativity difference for men among all ethno-racial categories. For women, however, the nativity difference among the MENA population was smaller (HR 0.71, CI 0.66–0.77), corresponding to 2.24 additional years of life expectancy for foreign-born MENA women. It is worth noting that other ethno-racial groups had variation in effect sizes between men and women, and the MENA differences are roughly comparable in magnitude.

Table 3 presents estimated life expectancies for each group, calculated from the Gompertz models as the average expected age of death conditional on living to age 65. Foreign-born MENA men had an e65 of 85.39 years, the highest life expectancy among foreign-born men, reflecting their large immigrant advantage. Similarly, foreign-born MENA women had the second highest life expectancy (e65 = 88.66) among foreign-born women. Notably, the life expectancies of foreign-born MENA men and women were both substantially higher than those of the foreign-born non-Hispanic White population in which they are often aggregated.

**Table 3:** Estimated life expectancy conditional on living to age 65 by group, sex, and nativity

	U.S.-born			Foreign-born		
	e65	e65 lower	e65 upper	e65	e65 lower	e65 upper
**Men**						
Non-Hispanic White	81.59	81.43	81.83	82.75	82.68	82.81
Asian and Pacific Islander	82.26	81.43	83.20	84.29	84.12	84.47
Non-Hispanic Black	79.67	79.38	80.13	80.06	79.81	80.31
Hispanic	82.07	81.41	82.89	84.50	84.34	84.66
MENA	82.26	80.40	84.33	85.39	85.00	85.78
Other	80.13	78.34	82.34	80.06	79.49	80.64
**Women**						
Non-Hispanic White	86.60	86.66	87.07	87.46	87.39	87.52
Asian and Pacific Islander	87.34	86.69	88.75	88.22	87.98	88.45
Non-Hispanic Black	85.25	85.31	86.28	88.64	88.16	89.12
Hispanic	87.05	86.51	88.17	89.65	89.46	89.85
MENA	86.42	84.28	89.02	88.66	88.17	89.16
Other	85.18	83.35	88.34	84.19	83.41	84.99

Life expectancy conditional on living to age 65 (e65) was calculated from truncated death distributions reflecting deaths that occurred between 1988 and 2005

## Discussion

This study provides one of the first national-level analyses of MENA American over-65 mortality outcomes. By developing a new machine-learning name classification algorithm, it offers a method for studying MENA American outcomes in the absence of other identifying information. Contrary to some state-focused studies of primarily Arab populations at younger ages, this analysis found evidence of an immigrant mortality advantage among the over-65 MENA population.

The methods developed in the study can be applied to other data sources with given name and surname information, such as medical records. Because MENA populations are formally classified as white in the United States, they are often “invisible” in such data sources. However, scholars have increasingly argued their social experiences do not fit this classification in ways that may affect their economic, social, and health outcomes [23–26]. Our results support this argument, as the MENA population outcomes were distinct from the non-Hispanic white population in which they are often aggregated.

There are limitations to relying on name-classification. Although the model fit statistics suggest it performed reasonably well in comparison to direct name matching, both Type I and Type II errors are possible. Many names in MENA countries may be common in other Muslim populations outside of the region. And although the method is likely effective at detecting U.S.-born MENA individuals with ethnically-distinct names, it would not be able to classify individuals with non-MENA names, which becomes more common with subsequent generations.

It is unclear why this study found an immigrant advantage among MENA populations where others have not, but there are a number of possible theoretical and methodological reasons worth exploring in future research. This is one of the few national-level examinations of MENA mortality, and studies that have primarily examined communities with high concentrations of Arab co-ethnics, particularly in Michigan and California, might miss patterns in other contexts. More than 68% of the records classified as MENA in the BUNMD data were from a state other than California or Michigan.

In addition, this study was limited to over-65 mortality, and the unique age, period, and cohort dynamics in this sample may result in different patterns compared to younger or more recent immigrant populations. The mean year of first social security application among the MENA records was 1983, suggesting this sample arrived among the earlier waves of post-1965 MENA immigration to the United States, which was a different composition from more recent arrivals, who come from a wider range of sending countries.^3^ In terms of period effects, this study’s coverage largely misses the post-2001 social environment, in which selection factors may have changed as more refugees from MENA countries made up the migration flows, and MENA populations also face more discrimination, marginalization, and related health risks.

## Supplementary Information

Below is the link to the electronic supplementary material.

Supplementary file1 (DOCX 46 KB)

## Data Availability

Data for this analysis is publicly available from the University of California-Berkeley’s CenSoc project: https://censoc.berkeley.edu/data/. Replication code is available on the SocArxiv open repository.

## Footnotes

1 Our model is an adaptation of this model, which was published in the “ethnicolr” Python package.

2 Please see the supplemental Appendix for full details about the LSTM model development and benchmarking.

3 See the Appendix for additional information about the composition of the MENA sample.
