# Supplementary material for: The Immigrant Mortality Advantage Among Over-65 Middle Eastern and North African Immigrants to the United States
Source: J Immigr Minor Health. 2024 Aug 4;26(6):977–83. doi: 10.1007/s10903-024-01622-5 (PMC11607005; doi:10.1007/s10903-024-01622-5)
Supplement: Supplementary file 1 — Supplementary file1 (DOCX 46 KB) [file 10903_2024_1622_MOESM1_ESM.docx]

**Appendix**

**A. Name classification algorithm**

In order to identify a likely U.S.-born MENA population for analysis, we trained a long short-term memory machine learning model to classify names based on character clusters within the names. Previous literature has similarly relied on probabilistic assessments of names in order to classify populations without identifying information, but the dominant approach has used direct-name matching to a list of names with known ethnic or national background information. This has the limitation of missing names without a direct match, resulting in a smaller and possibly less-representative sample (see below for a replication of our analysis using the direct-matching method).

We instead trained a name-classification model on a subset of foreign-born records in the BUNMD data, and we used the trained model to subsequently classify U.S-born records with similar names. Foreign-born MENA records were identifiable in the BUNMD data based on place of birth. The following countries were included in the list of MENA countries for inclusion: Afghanistan, Algeria, Bahrain, Egypt, Gaza Strip/West Bank, Iran, Iraq, Israel, Jordan, Kuwait, Lebanon, Libya, Morocco, Oman, Saudi Arabia, Sudan, Syria, Tunisia, United Arab Emirates, and Yemen. The name of the mother and father were treated as having the same birth country and added to the list of names to create a larger training dataset. This resulted in 2,590,329 total training names, of which 82,476 were classified as MENA. A subset of individuals born in South Asia was also included as a separate category to help the model distinguish the two nearby regions.

Our model combines each given name and surname in the BUNMD records and then encodes the name using byte pair encoding. This method begins by tokenizing the names as single-character representations, and it then combines adjacent pairings that frequently occur in the data.^[[1]](#footnote-1)^ For instance, a common character cluster may be combined into a token, such as the “kh” consonant that is common in Arabic and Persian language names. Longer tokens may also be created, such as “Abdul” or multi-character subwords that are common across a variety of names.

The training dataset was used to train a Long Short Term Memory (LSTM) model, which we adapted from the Python ethnicolr package.^[[2]](#footnote-2)^ The LSTM model is an effective machine learning approach for analyzing patterns in sequences and is often used for detecting temporal ordering, such as time series events or natural language processing.^[[3]](#footnote-3)^ The model was trained on token windows of 25, with a batch size of 32 and 12 epochs. This model allows for detecting both the presence and sequence of character and subname clusters in names unique to the MENA group. Some character clusters are unique to Arabic and Persian alphabets, and can indicate a higher likelihood of MENA background by their presence alone (e.g., “kh”). The LSTM model's ability to detect sequences of character patterns should further return patterns in combinations of characters, which is useful for picking up on spelling variations that are similar but not exact. For instance, the same origin name might include different variations of the same underlying bigrams and similar sequences, such as: Abdallah, Abdellah, Abdollah, Abdullah, Abdulla, Abdalla.

The name list was divided into a training set of an 80% sample of the names. Table A1 presents evidence of the model effectiveness on the remaining 20% test data set. For MENA-classified records, precision was .86, recall was .79, and the overall F1 score was .82. Although the model was not as effective for predicting other ethno-racial categories, particularly foreign-born non-Hispanic Black names, the LSTM model was only used for MENA classification in our analysis. The lower Recall indicates more “false negatives,” however a posthoc examination of missed MENA-classified names suggests a substantial number of records with birthplace in MENA countries but common European-origin names. Given the history of European colonialism in the region and birth years that include the late 19^th^ and early 20^th^ centuries, it appears that some of the records include European-ancestry individuals who were born in a MENA country and subsequently migrated to the United States. In this case, the Recall performance for name ethnicity may be better than suggested by the metric.

**Table A1. Out of Sample Performance for LSTM Name-Matching Algorithm**

|  | Support | Precision | Recall | F1 |
| --- | --- | --- | --- | --- |
| Asian | 106462 | 0.94 | 0.85 | 0.89 |
| Black | 31329 | 0.76 | 0.66 | 0.71 |
| Hispanic | 164939 | 0.87 | 0.94 | 0.91 |
| MENA | 16495 | 0.86 | 0.79 | 0.82 |
| South Asian | 12892 | 0.88 | 0.88 | 0.88 |
| White | 185949 | 0.88 | 0.89 | 0.89 |
| Weighted average | 518066 | 0.88 | 0.88 | 0.88 |

The trained algorithm was then used to predict ethno-racial group for each of the 4,401,173 records in the BUNMD subsample that fit our criteria for analysis. In cases where the algorithm did not predict a name was likely of MENA origin, or if the original ethno-racial category was non-Hispanic Black (due to the higher prevalence of Muslim-origin names among this group), the original ethno-racial classification from the BUNMD race variable was used.

As shown in Table A2, approximately 85% of records that were re-classified as MENA were originally identified as non-Hispanic White, and an additional 13% were previously classified as Asian.

**Table A2. Previous Racial Classification of MENA-Classified Records**

| Original racial classification | n | Proportion of total |
| --- | --- | --- |
| Non-Hispanic White | 24,259 | 0.85 |
| Other | 256 | 0.01 |
| Asian | 3,632 | 0.13 |
| Hispanic | 280 | 0.01 |

*Note: Individuals who were originally classified as non-Hispanic Black kept their original racial classification in order to reduce misclassification of non-MENA Muslim names.*

In the main manuscript, we provide the results of life expectancy calculations using the new ethno-racial group variable. Although the MENA population is relatively small, classifying MENA names in the BUNMD records not only provides information on MENA life expectancy patterns, but it also changes the composition of the ethno-racial groups in which they were previously classified. Table A3 presents life expectancy calculations for the original ethno-racial variable in the BUNMD data. The differences are relatively small, with life expectancies at 65 only about 0.2-0.3 years lower for White and Asian groups after disaggregating the MENA category Notably, the life expectancy for the “Other” category was substantially higher for both men and women after removing identified MENA names.

| **Table A3. Estimated Life Expectancy Conditional on Living to Age 65 by Original Ethno-Racial Classification, Sex, and Nativity** | | | | | | | |
| --- | --- | --- | --- | --- | --- | --- | --- |
|  | U.S.-Born | | |  | Foreign-Born | | |
|  | e65 | e65 lower | e65 upper |  | e65 | e65 lower | e65 upper |
| **Men** |  |  |  |  |  |  |  |
| White | 81.63 | 81.44 | 81.83 |  | 82.90 | 82.84 | 82.96 |
| Asian | 82.27 | 81.41 | 83.15 |  | 84.37 | 84.20 | 84.55 |
| Black | 79.75 | 79.38 | 80.13 |  | 80.14 | 79.89 | 80.39 |
| Hispanic | 82.14 | 81.41 | 82.89 |  | 84.57 | 84.41 | 84.73 |
| Other | 77.27 | 73.14 | 83.29 |  | 79.71 | 78.50 | 80.98 |
| **Women** |  |  |  |  |  |  |  |
| White | 86.87 | 86.67 | 87.07 |  | 87.74 | 87.68 | 87.81 |
| Asian | 87.70 | 86.68 | 88.72 |  | 88.59 | 88.36 | 88.83 |
| Black | 85.79 | 85.31 | 86.28 |  | 89.18 | 88.70 | 89.66 |
| Hispanic | 87.34 | 86.52 | 88.18 |  | 89.94 | 89.74 | 90.13 |
| Other | 82.00 | 76.81 | 88.58 |  | 83.51 | 82.00 | 85.06 |

*Note: Life expectancy conditional on living to age 65 (e65) was calculated from truncated death distributions reflecting deaths that occurred between 1988 and 2005.*

**B. OLS analysis**

As an alternative to the Gompertz truncated hazard model, we also tested our results using ordinary least squares regression. Following recommendations from Goldstein and Breen^[[4]](#footnote-4)^, we include a birth year fixed effect to account for left and right truncation of the death distributions. However, they argue that this method is likely produce coefficients that are biased toward zero.

Table A2 presents results from our OLS models. The results are substantively similar to the Gompertz survival models, although there are some notable differences. As with the main results, the “immigrant advantage” is smaller among MENA women (b = 0.81) compared to MENA men (b = 1.19). The relative effect sizes are also smaller across groups, perhaps indicating biased estimates due to the double truncation of the mortality curves.

**Table B1. OLS Regression Analysis of Age at Death By Nativity and Group**

|  |  | Men | | Women | |
| --- | --- | --- | --- | --- | --- |
|  |  | Estimate | SE | Estimate | SE |
| White | Intercept | 94.66 | 0.05 | 95.54 | 0.05 |
|  | Nativity effect | 0.64 | 0.01 | 0.49 | 0.01 |
| Asian | Intercept | 94.65 | 0.26 | 95.37 | 0.30 |
|  | Nativity effect | 0.94 | 0.03 | 0.39 | 0.04 |
| Black | Intercept | 94.96 | 0.10 | 95.68 | 0.11 |
|  | Nativity effect | 1.26 | 0.05 | 1.10 | 0.04 |
| Hispanic | Intercept | 94.02 | 0.23 | 95.52 | 0.30 |
|  | Nativity effect | 0.94 | 0.02 | 0.85 | 0.02 |
| MENA | Intercept | 94.91 | 0.58 | 96.06 | 0.71 |
|  | Nativity effect | 1.19 | 0.07 | 0.81 | 0.08 |
| Other | Intercept | 95.17 | 0.54 | 95.85 | 0.71 |
|  | Nativity effect | -0.08 | 0.10 | -0.16 | 0.11 |

*Note: Regression models include dummy variables for birth year.*

**C. Direct-matching method**

We re-calculated the main results using a direct name-matching method to classify MENA records, as an alternative to the machine-learning approach. This method has been used previously to identify MENA populations by matching names in records with an external list of names with known MENA ancestry. We used a list of names provided by the Social Security Administration that includes country of origin information for foreign-born individuals who submitted a social security application. This is arguably one of the most nationally-representative and largest name lists given its source from adminsitrative records. For each given name and surname, its occurrences among MENA immigrants are calculated relative to the total number of occurrences, providing a way to probabilistically classify name combinations. Names were included if they had at least five occurrences associated with at least one of MENA-origin country. The proportional representation of each name was calculated, and the resulting source file included 40,450 given names and 45,939 surnames. Following similar studies, a positive match was identified if the combination of scores for the given name and surname was higher than .15.

The direct-matching method resulted in a substantially smaller sample of MENA records. Many names that were classified as MENA by the LSTM algorithm did not have a direct match in the source name list. Of the total 4,401,173 names in the final dataset, 409,465 did not have a direct match for both the first or last name, and more than 63% were missing a match for one of the names.

| **Table C1. Performance of Name Classification Methods on BUNMD Subset Born in a MENA Country** | | |
| --- | --- | --- |
|  | Direct Matching Method | Machine Learning Method |
| Precision | 0.50 | 0.86 |
| Recall | 0.31 | 0.78 |
| F1-Score | 0.38 | 0.82 |

*Note: Evaluation metrics are calculated on a subset of individuals born in a MENA country and differ from similar metrics from a smaller subset of test data in Table A1.*

This resulted in only 19,858 MENA records using the direct-matching method, compared to 28,427 in the main results. Because both methods relied on country of birth for foreign-born MENA records, the U.S.-born comparison is more revealing of the differences. The direct-matching method resulted in only 2,308 matches for U.S.-born records, compared to 11,036 using the LSTM model. As shown in Table C1, evaluation metrics on the subset of foreign-born names were substantially lower using the direct-matching method, due to the large number of missing matches.

Table C2 presents the results of the Gompertz MLE analysis using this direct-matching method and corresponds to Table 2 in the main manuscript. The results are substantively similar, suggesting the direct-matching sample may be representative even though smaller. The nativity effect on e65 for MENA men in Table C2 of 3.71 is comparable to the 3.13 years in Table 2, and in both tables it is the largest effect among men. The effect for women of 1.76 years is slightly lower than the 2.24 years in Table 2.

**Table C2. Gompertz MLE Analysis Using Direct-Matching to Identify MENA Names**

|  | Hazard Ratio | | |  | Effect on e65 | | |
| --- | --- | --- | --- | --- | --- | --- | --- |
|  | Estimate | Lower | Upper |  | Estimate | Lower | Upper |
| **Men** |  |  |  |  |  |  |  |
| White | 0.85 | 0.84 | 0.86 |  | 1.16 | 1.10 | 1.23 |
| Asian | 0.75 | 0.73 | 0.77 |  | 2.05 | 1.87 | 2.22 |
| Black | 0.95 | 0.92 | 0.98 |  | 0.39 | 0.14 | 0.65 |
| Hispanic | 0.72 | 0.70 | 0.73 |  | 2.43 | 2.27 | 2.59 |
| MENA | 0.58 | 0.53 | 0.64 |  | 3.71 | 3.07 | 4.35 |
| Other | 1.01 | 0.94 | 1.09 |  | -0.10 | -0.67 | 0.48 |
| **Women** |  |  |  |  |  |  |  |
| White | 0.88 | 0.87 | 0.89 |  | 0.86 | 0.79 | 0.92 |
| Asian | 0.87 | 0.84 | 0.91 |  | 0.90 | 0.66 | 1.13 |
| Black | 0.64 | 0.60 | 0.68 |  | 3.45 | 2.96 | 3.94 |
| Hispanic | 0.67 | 0.65 | 0.69 |  | 2.61 | 2.42 | 2.81 |
| MENA | 0.76 | 0.69 | 0.84 |  | 1.76 | 1.14 | 2.39 |
| Other | 1.14 | 1.03 | 1.27 |  | -1.01 | -1.80 | -0.22 |

*Note: Estimates reflect the difference between foreign-born and U.S.-born individuals within each ethno-racial group. The first three columns estimate the hazard ratio of foreign-born nativity status relative to U.S.-born. The last three columns convert this difference into an estimated effect on life expectancy conditional on living to age 65.*

**D. Sample Composition**

As noted in the main manuscript, the sample of MENA foreign-born and U.S.-born individuals in this study is not representative of the full MENA population in the United States. Because analysis is limited to over-65 mortality, the sample over-represents earlier waves of migration from the Middle East and North Africa, which differs from contemporary migration. The below figure shows the number of foreign-born individuals from each country in the MENA sample. The largest group by far is from Iran, followed by Lebanon, Egypt, and Syria.

Although the BUNMD data does not contain information on year of immigration, data on the year of first Social Security application can provide an approximation of many immigrants’ naturalization timelines. Among the foreign-born MENA sample, the mean year of first SSA application was 1983, and the vast majority of the sample fell between the late 1970s and late 1980s.

| **Figure D1. Country of Origin Among MENA Immigrants in BUNMD Sample** |
| --- |
|  |
|  |

1. 1. Shibata Y, Kida T, Fukamachi S, Takeda M, Shinohara A, Shinohara T, et al. Byte Pair encoding: A text compression scheme that accelerates pattern matching. 1999; [↑](#footnote-ref-1)
2. Sood G, Laohaprapanon S. Predicting Race and Ethnicity From the Sequence of Characters in a Name. ArXiv180502109 Stat. 2018; Available from: http://arxiv.org/abs/1805.02109 [↑](#footnote-ref-2)
3. Van Houdt G, Mosquera C, Nápoles G. A review on the long short-term memory model. Artif Intell Rev. 2020;53:5929–55. [↑](#footnote-ref-3)
4. Breen CF, Goldstein JR. Berkeley Unified Numident Mortality Database: Public administrative records for individual-level mortality research. Demogr Res. Max-Planck-Gesellschaft zur Foerderung der Wissenschaften; 2022;47:111–42. [↑](#footnote-ref-4)
